# Supplementary figures and images for: Gene editing of PKLR gene in human hematopoietic progenitors through 5’ and 3’ UTR modified TALEN mRNA
Source: PLoS One. 2019 Oct 16;14(10):e0223775. doi: 10.1371/journal.pone.0223775 (PMC6795450; doi:10.1371/journal.pone.0223775)

**A**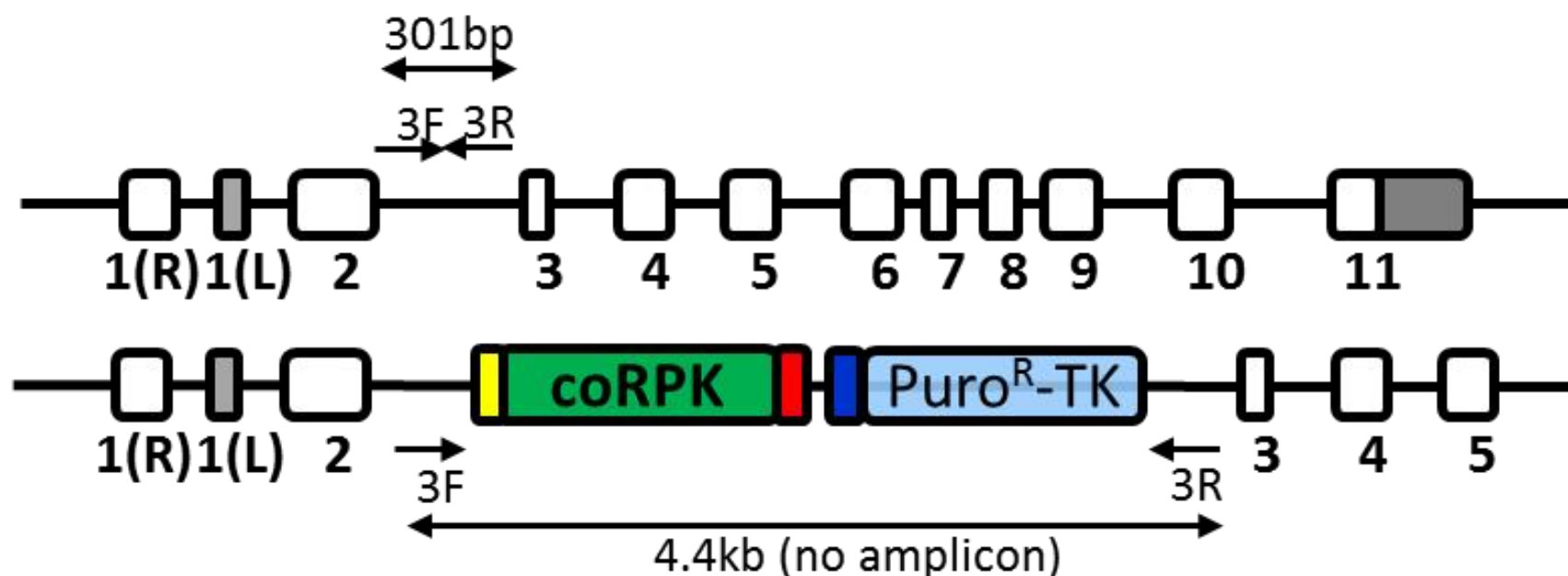**B**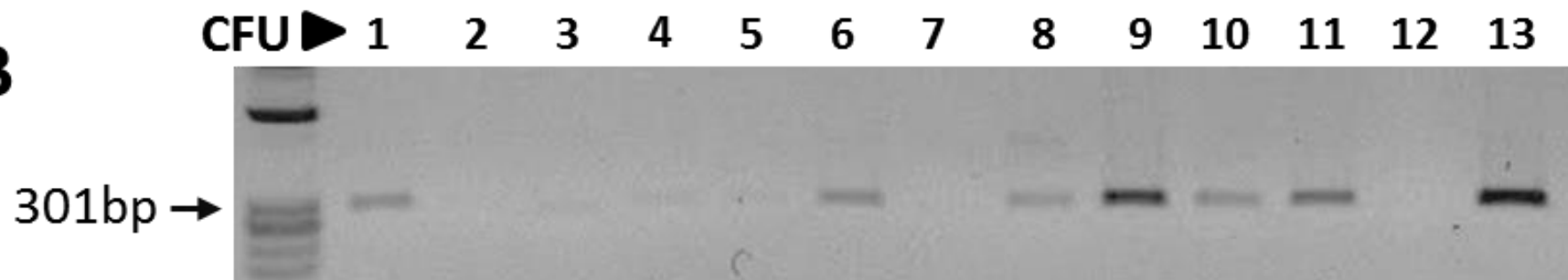

Supplement: S1 Fig — (A) Diagram of PCR analysis. Positive CFU for nested PCR were re-evaluated to test if the integration of our matrix had occurred in one allele or in both alleles. (B) Representative data from one of the independent experiments shown the analysis of monoallelic/biallelic gene editing of PKLR locus in human hematopoietic progenitors. Positive bands meant single allele integration, and no band indicated biallelic integration. (PDF) [file pone.0223775.s001.pdf]

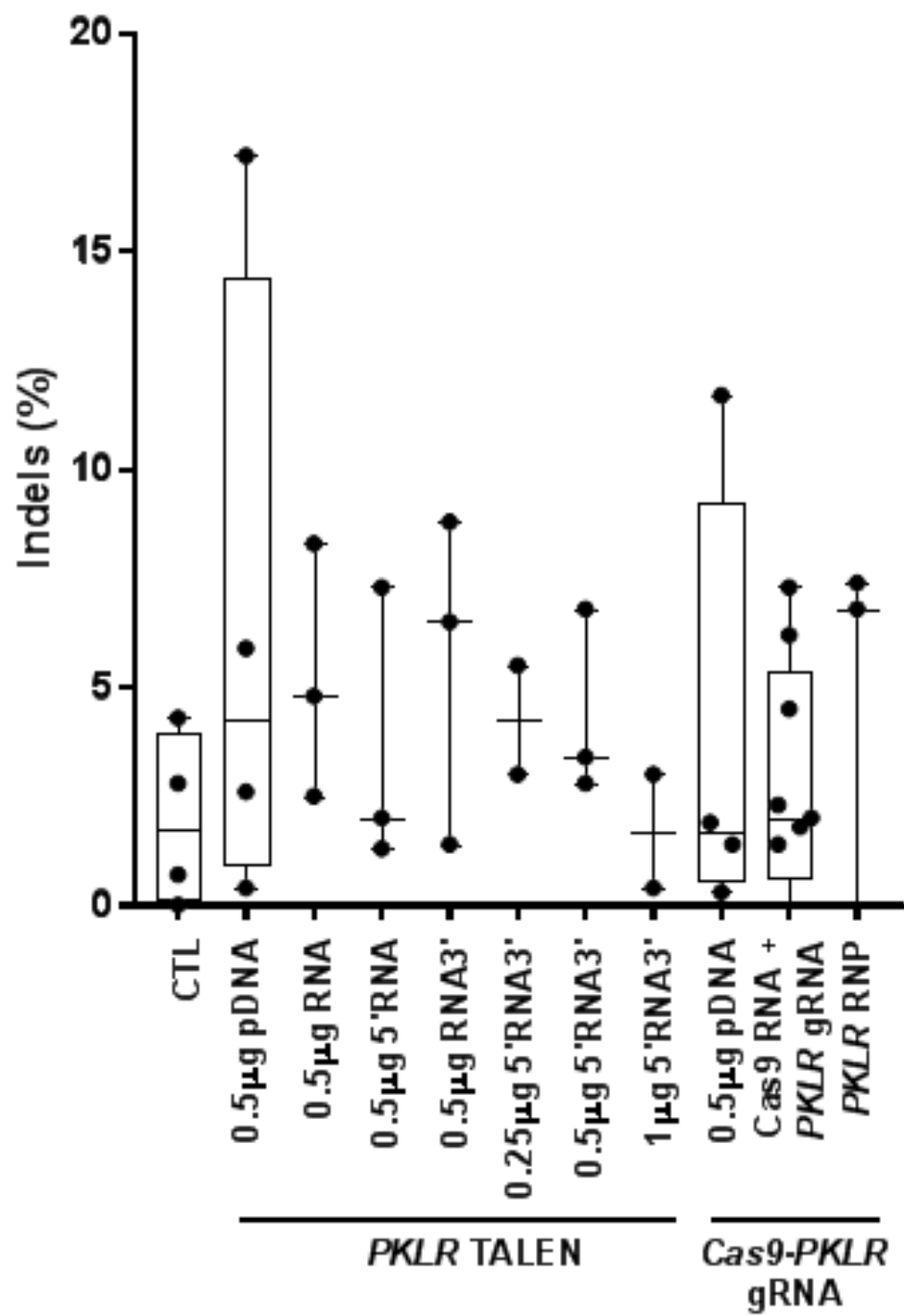

Supplement: S2 Fig — Used amount of each reagent is indicated. Data from four independent experiments. (PDF) [file pone.0223775.s002.pdf]
