# Supplementary material for: Gene editing of PKLR gene in human hematopoietic progenitors through 5’ and 3’ UTR modified TALEN mRNA
Source: PLoS One. 2019 Oct 16;14(10):e0223775. doi: 10.1371/journal.pone.0223775 (PMC6795450; doi:10.1371/journal.pone.0223775)
Supplement: Attachment — Submitted filename: Quintana-Bustamante_al_Response to Reviewers_rev_B.docx [file pone.0223775.s003.docx]

Oscar Quintana-Bustamante

Differentiation and Cytometry Unit

CIEMAT/CIBERER/IIS-FJD

E-mail: oscar.quintana@ciemat.es

**RE: PONE-D-19-14838**	August 27^th^, 2019

Dear Editor,

Thank you very much for reviewing our manuscript and for allowing us to present a revised version. In this revised version we have addressed the comments and suggestions performed by the reviewer. Please find enclosed the revised version of our manuscript entitled **"Gene editing of *PKLR* gene in human hematopoietic progenitors through 5’ and 3’ UTR modified TALEN mRNA ".** All the modifications have been highlighted in the manuscript.

Below, you will find a detailed description of the modifications and clarifications we have included in the manuscript to answer the comments and suggestions provided by the reviewer. We very much hope that, with these clarifications, the manuscript could be acceptable to be published in Plos one.

Yours sincerely,

Oscar Quintana-Bustamante

**Reviewer #1**:

This manuscript reported a feasible way, although inefficient, to edit *PKLR* gene in HPSCs, which may be clinically applicable to correct PKD in the future after further improvements. Through a thorough reading and reviewing, I think this manuscript can be accepted after minor revision. The main questions in this manuscript to my point of view are as follows.

1. Abbreviations (e.g. RPK and PKLR) should be explained at the first occurrence in the text.

We have added the full name of RPK (R-Type Pyruvate Kinase) and *PKLR* gene (Pyruvate Kinase L/R gene) at the first occurrence in the text (see page 3).

3. According to the supplementary data, several figures in the first paragraph of page 13 seem to be miscalculated: 56% should be 53.8%, while 44% should be 46%. In addition, the source of the data for “5.8 knock-in CFUs out of 1000 initial CBCD34+ cells” could not be found.

Although we showed a representative picture of the agarose gel from a single experiment, the percentages were calculated from different experiments. To clarify this point, we have explained this in the main text (first paragraph of page 13) and in the supporting information 1 caption (page 33).

Additionally, the data “5.8 knock-in CFUs out of 1000 initial CB-CD34^+^ cells” has been reviewed and modified in the text (first paragraph page 14). We have modified “5.8 knock-in CFUs out of 1000 initial CB-CD34^+^ cells” for “5.8 knock-in CFUs out of 10,000 in the absence of puromycin selection CFUs”. This data has been calculated taking into account the number of CFUs from CD34+ nucleofected with TM with puromycin treatment (Figure 1D) in comparison with CFU derived from CD34+ nucleofected with TM without puromycin selection (Figure 1C). Nevertheless, this data can be also expressed in terms of total CD34+ cells. Consequently, we detected 16.3 knocked-in CFUs out of one million initial CB-CD34+ cells (average of three independent experiments).

5. In Results part, the authors declare that the use of dmPGE2 did not improve the efficiency of the specific integration. However, it seems cursory to draw this conclusion just based on 4 CFUs. To support this conclusion, more puromycin-selected colonies are needs for further analysis although the dmPGE2 treatment will do harm to hCD34+ cells and make it hard to yield colonies.

We claimed dmPGE_2_ treatment did not improve the efficiency of the specific integration taking into account our data of CFUs and of the human engraftment in NSG mice. As the reviewer pointed out, the toxicity observed in the CFUs along all the procedure (nucleofection, Puro selection and dmPGE_2_ treatment over all the culture time) is high. With these conditions, only 1 out of 4 CFUs were positive for Homologous Direct Repair (HDR), which is lower than in absence of dmPGE_2_ (15 CFUs out of 21) (Table 3 and figure 6). An additional experiment gave rise to an uncountable number of human hematopoietic progenitors. A PCR analysis of the bulk culture was also negative, reinforcing the data obtained. We didn’t include this last experiment because the information was not quantitative. These results might indicate that dmPGE_2_ is toxic for CFUs that have undergone nucleofection and selection with Puromycin, and even detrimental to enrich HDR in CFUs with puromycin. Additionally, this detrimental effect of the dmPGE_2_ to select HDR HSPCs with puromycin was also observed when transplanting human cells after gene editing in NSG. Hence, human engraftment of the gene edited samples in presence of dmPGE_2_ transplanted in NSG mice was higher than in the animals transplanted with CB-CD34^+^ cells without dmPGE_2_ treatment, although no gene edited population could be detected in the progenitors present in the human engraftment. Somehow, the addition of dmPGE_2_ rescued the HDR-negative HSPCs from puromycin selection, favoring the human hematopoietic engraftment of these rescued (and non-edited) HSPCs, as previously described by Hoggart et al 2009 and Pelus et al 2011, without incrementing the frequency of HSPCs with HDR. We have added a sentence in the Discussion, (from line 600 to 606) to point out the possible interference of dmPGE_2_ in puromycin selection.

6. The figure legends in the text are too simple to understand. It is important to provide enough information so that the readers can easily understand the content of article. For example, what does the λ in Figure 2A and IX in Figure 3B mean? Are they DNA markers or something else?

We have expanded the information in the figure legends more deeply and we have described these two DNA markers (λ and IX) to clarify the information of the figures.

**Reviewer #2**:

Overview


This manuscript outlines a study using CRISPR- and TALEN-directed gene editing to repair mutations in the *PKLR* gene, which produces a pyruvate kinase, mutations within which are responsible for pyruvate kinase deficiency (PKD). This rare metabolic disease has been a target of previous gene editing protocols but the overall outcome was disappointing sense a low yield of corrected cells was attained. As has been seen for other gene editing protocols, attempts to increase the gene repair efficiency led to a significant level of toxicity. The authors now extend the work by comparing several programmable nucleases and their associated systems coupled to a more clear definition of expansion and selection time. The results are somewhat disappointing but not truly unexpected, since many ex vivo approaches, designed with the best of intentions, lead to a very low level of gene edited HPSCs following engraftment. The authors conclude that they have demonstrated the feasibility of gene editing of the PKLR gene yet further improvements must be made in order to achieve clinical application.

Critique


Overall, this is a standard targeting approach to a metabolic disease, a group of diseases that have a myriad of mutations possibly approachable by gene editing. What sounds so simple is so hard because it's not just about the gene editing, which likely works with high efficiency and specificity now, but rather about the treatment of the cells before and after the reaction. These are well-established authors who have a good track record of publishing solid work and this is no exception. I would suggest some modifications to the manuscript particularly in terms of detail. I think it's also important that the authors develop a stronger argument as to why their work is worth publishing even though for the overall scientific community, they do provide some important information. Just showing that gene editing in *PKLR* is feasible in a new cell type, is really not meeting the high bar now set for publication in significant journals. My recommendation is to accept the paper with major revision following the points below:


On page 5, in the first section of the Methods and Materials, the authors indicate that they are growing CD34+ cells in antibiotics. While it's clear that using puromycin as a selection is helpful experimentally, this will be problematic in a transition to *ex vivo* gene editing protocols. Antibiotic treatment of any cells causes major redistribution of metabolic pathways including that associated with gene editing. Why did they choose to do so as it is not usually a good idea? A strong argument needs to be made as to how this would impact clinical application only because their previous work have moved along this path already. This is important points of the will likely affect the outcome of their experiments and the generalization of their experiments into clinical application. It's also important to define the concentration of puromycin in more detail.

We agree with the reviewer point of view regarding the concerns of the clinical application of puromycin resistence. Considering the high efficacy of gene editing reached in HSPCs by others, it could be considered we considered that no selection would be required. On the other hand, the same contributions also claim that some kind of selection would be required in settings where a high proportion of corrected cells is required. Thus, we tested puromycin as a potential alternative. Puromycin selection enriched gene editing CFUs from 5.8 knocked-in CFUs out of 10,000 total CFUs (from line 289 to line291) to over 7 knocked-in CFUs out of 10 puromycin resistant CFUs, which is around 1,000-fold HDR enrichment in CFUs. Consequently the use of this or any other selection system, such as expression of truncated NFGR, might help to increase the number of gene editing events when gene editing frequency is under the therapeutic needs. In addition, as it is described in “Gene editing tools” (Material and Methods, Page 5, lines 115-117), the donor matrix has an expression cassette formed by the PGK promoter driving the expression of the Puromycin resistance and Tymidine Kinase fusion protein, and the bGH PolyA signal, and flanked by LoxP sequences. Consequently, TK suicide gene might be used to eliminate knocked-in cells if any hypothetical problem occurred in the clinical use of our approach.

Moreover, puromycin selection was performed 4 or 6 days after nucleofection of CD34+ cells (see Figure 1B and Material and Methods lines 150-151), when gene editing was expected to be occurred. So, we do not think that the addition of puromycin in the CD34+ culture could have an impact on gene editing since it should have already occurred.

The concentration of puromycin (1µg/ml) used was added at line 151.

The RNP is a logical choice for use in any sort of ex vivo application but it appears out of nowhere and so what concentrations were used and why were they chosen? The RNP has a rapid turnover in the cell and in the nucleus and in some cases does not actually reach the nucleus.

We completely agree with this Reviewer’s comment. We have added the amount of RNP used in the experiments (see lines from 131 to 135) according with IDT or PNA Bio companies, and the concentrations of RNP was similar to the ones described by Modarai et al (see next comment). Additionally, we used Amaxa system, which allows the nucleofection of gene editing tools into the nucleus, as Modarai et al. has previously reported. We have included also this reference in the Discussion. Furthermore, we have performed three new independent experiments assesing the nuclease activity of RNP in comparison with TALEN delivered as plasmid DNA, and TIDE analysis reveals that the percentage of indels after nucleofection of either gene editing tools into CB-CD34+ cells is very similar (see lines from 353 to 357 and new figure 4C).

While I do not follow this exact field, I did notice several papers appearing Molecular Therapy (NA) last year that detailed the challenges with delivery of ex vivo therapy (Modarai et al). I often utilize this paper as an example of how the details are important and that transfection efficiency can often modify/slant outcomes... I would reference this paper and discuss how the authors overcome this important experimental parameter.

We thank Reviewer’ suggestion and we have included and discussed Modarai et al. publications. We have used the nucleofection system from Amaxa, which is able to deliver DNA into cell nucleus, as it is shown in Modarai et al. The rest of the conditions described by Modarai were very similar to ours. We have used a range of RNP from 0.2μM (3.3μg Cas9+ 6μg SG) to 0.4μM (6.6μg Cas9+ 12μg SG), which matches with the ones used by Modarai. We agree with the conclusions of this straightforward article, the characteristic of the HSPCs used can make a difference since they used bone marrow CD34+ and we used CB-CD34+, and as they claimed the properties between both HSPCs are slightly different, especially the cell cycle status and the proportion of primitive HSPCs.

Additionally, as it is said by Modarai et al, cell viability might be affected by nucleofection of gene editing tools. According to our data, the nucleofection of DNA plasmids reduces the HSPC viability (see figure 1C and 3C). We replaced *PKLR* TALEN format (from DNA plasmids to modified mRNAs) reducing its toxicity but maintaining its gene editing efficacy (Figure 4B-C). Moreover, very little gene editing HSPCs but still detectable was observed after HSCT in immunodeficient mice. Therefore, the toxicity associated to the nucleofection of gene editing tools in primitive HSPCs was also reduced. However, our system is based in nucleofection of a nuclease (as DNA plasmid, mRNA) together with a donor matrix as DNA plasmid, which might reduce the success of our approach. We think that moving this donor matrix from a DNA plasmid format to an AAV format might help to improve even further both cell viability and gene editing efficacy. This last point is something that we are exploring nowadays.

What is the role of dmPGE(2)? (page 7).

As previously Hoggart et al 2009, Pelus et al 2011 and Genovese et al 2014 described, dmPGE2 enhances survival, preserver better primitive HSPCs and their engraftment ability of HSPCs. We has supplemented the culture with dmPGE2 to reduce the toxicity associated with nucleofection and puromycin selection and to promote the engraftment of gene editing primitive HSPCs. Unexpectedly, gene editing efficiency was shrunk as well as CFU efficiency. Although we do not know the exact cause, we speculate that dmPGE2 was detrimental to select gene editing HSPCs, favoring an overall survival, which resulted in a reduction in the gene editing frequency. This point is discussed deeper in Discussion (see lines from 587 to 598).

While this is not the author’s problem, it's now widely accepted that the Surveyor Assay does not sufficiently reflect CRISPR activity in human cells. And, while the equation used to develop the percentage of cleavage is useful, this assay misses small indels less than 3 to 4 bases.... DNA sequence analysis and TIDE must be used in parallel even in the early sections of the work.

We have included a new analysis to compare the gene editing efficacy of PKLR TALEN and RNP assessed by TIDE software, following the helpful Reviewer’ suggestion. It is included in Results section (in lines 353-357 and in figure 4C) and in Materials and Methods (lines 184-189). We have tested the newest gene editing tools in comparison to PKLR TALEN and no significant differences were observed in terms of indels generation. This fact is also discussed in lines 537 to 550.

The authors need to explain why they do not see any heterozygotes? I'm actually a bit surprised by this as one would expect to see a small percentage of them.

We addressed this Reviewer’s question at lines from line 280 to line 288 and Supporting information 1. We have expanded this part to clarify the frequency of HDR positive heterozygous of the gene edited CFUs. Indeed, 46% of gene editing CFUs showed the HDR in one allele, and 53.8% in both alleles, as the Reviewer has expected.

On page 13, the authors loosely use the term homologous recombination.... It is now true that gene editing has become a garbage heap of terms, mostly inappropriate, the authors need to be quite careful in using the term homologous recombination. The basket term is now considered homology-directed repair since homologous recombination is reserved for chromosomal crossovers that occurr during meiosis and, less often, in mitosis. It's not a big point, but it's a bit sloppy.

Following Reviewer’ suggestion, we have substitute “homologous recombination” for “homology-directed repair (HDR)” in different parts of the text.

I'm a little surprised about the toxicity associated with DNA nucleofection since much of the rest of the world believes that nucleofection is much gentler than electroporation.... Please explain.

We agree with the Reviewer in this point. Although nucleofection is the less harmful alternative to introduce DNA into cells, we and others have seen that toxicity in human HSPCs is DNA dose-dependent. As it is shown in Figure 1C, the nucleofection of *PKLR* TALEN plasmids and donor matrix, in comparison with only donor matrix, impacts on CFU number negatively. Additionally, DNA-toxicity was observed over time as it is described from line 335 to 338 Similarly, the nucleofection of different doses of PKLR TALEN mRNAs are much less harmful than PKLR TALEN DNA plasmids nucleofection (Figure 3C). We think this is one of the key points of our results. DNA nucleofection harms human HSPCs and reduces CFU numbers (Figure 1C and 3C) diminishing their engrafting capacity (Figure 6B). Consequently, the substitution of any gene editing tool in a DNA format should be considered in a clinical setting. Indeed, the gene editing field has already moved in this direction since nucleases are being used as mRNA or RNP and donor matrix as AAVs. To reflect this Reviewer’s consideration, we have strengthened this point in the text (see lines 535 and 547 of the Discussion).

On page 16, this goes back to my point about referencing previous data that has already established. i.e. lack of activity in expression constructs as opposed to the RNP. While it is true that senior investigators such as this group have made important contributions and often reflect on their own work, there's no use reinventing the wheel. I think of a simple scan of the literature might be helpful to avoid experimental activity whose outcome is quite predictable, and has been previously reported.

We agree with Reviewer about the preferential use of RNP nowadays for primary cells, mainly because their activity i limited in time. However, to force the nuclease activity, we decided to use an all-in-one plasmid to express Cas9 and *PKLR* sgRNA for a long period of time. When we used RNP directed against the same genomic site as the TALENs, we didn’t observe significant differences in indels generation (see new figure 4C and lines 353-357).

Additionally, we could not see any improvement in HDR in a human cell line (figure 4D), This last point was unexpected since the use of CRISPR/Cas9 system seems to have displaced the use of other nucleases, such as TALEN and reinforces the concept stating that if a nuclease is good enough, no matter it is a TALEN a ZFN or a CRISPR, it can be very helpful in the improving gene editing.

Page 17, once again, the trend outlined in Table 2 has been previously reported... And needs to be cited.
A good place to talk about the delivery variations is in the Disucssion... Previous information, I suggest on page 23 at the bottom continuing on to page 24.

We have added these considerations on page 24.
